# Harnessing Cancer Stem Cells and 3D Organoids in Unravelling Spatial and Cellular Heterogeneity in Cancer

**DOI:** 10.3390/ijms27093790

**Published:** 2026-04-24

**Authors:** Eunsong Kwak, Haneul Kim, Eunhye Kim

**Affiliations:** Laboratory of Molecular Diagnostics and Cell Biology (MolCellBio), College of Veterinary Medicine, Gyeongsang National University, 501 Jinjudae-ro, Jinju 52828, Republic of Korea; kes7427@naver.com (E.K.); skyk@gnu.ac.kr (H.K.)

**Keywords:** cancer stem cells (CSCs), tumor heterogeneity, tumor microenvironment (TME), organoids, patient-derived organoids (PDOs)

## Abstract

Cancer exhibits pronounced heterogeneity at both spatial and cellular levels, contributing to variability in therapeutic responses and the emergence of treatment resistance. This heterogeneity is underscored by the diverse genetic, epigenetic, and phenotypic variations found within tumor cell populations. Cancer stem cells (CSCs), although representing a minor fraction of tumor cells, possess the capacity to self-renew and differentiate, thereby driving the dynamic evolution of tumor heterogeneity. CSCs interact intricately with various elements of the tumor microenvironment (TME), further amplifying this heterogeneity. Recent advancements in organoid technology have facilitated the development of CSC-derived organoid models that more faithfully recapitulate the TME and intratumoral heterogeneity, which conventional 2D culture systems fail to replicate. These CSC-derived organoid systems not only preserve the structural and genomic characteristics of tumors, but they also enable the exploration and evaluation of therapeutic strategies that reflect tumor complexity. However, CSC-derived organoid systems face several challenges, such as the rarity of CSCs, lack of standardized culture conditions, absence of TME components, limited predictive accuracy, and insufficient modeling of tumor heterogeneity. This review discusses these limitations and explores potential solutions, including the use of artificial intelligence (AI) to enhance treatment predictability. These innovations may improve the utility of organoid models for therapeutic evaluation and for targeting tumor heterogeneity. Ultimately, CSC-derived organoids may serve as a valuable platform for advancing precision medicine and cancer research.

## 1. Introduction

Understanding cancer is, in essence, understanding its heterogeneity. Intra-tumoral heterogeneity, observed at both spatial and cellular levels, is a major barrier to effective cancer treatment [1,2,3]. In addition to genetic diversity, tumor heterogeneity is increasingly recognized as a dynamic process driven by cellular plasticity and evolutionary adaptation [4]. Cancer cells continuously undergo phenotypic transitions in response to environmental pressures, contributing to clonal selection and therapeutic resistance [5]. Among the contributors to this complexity, cancer stem cells (CSCs), characterized by their capacity for self-renewal and therapy resistance, have emerged as key drivers of tumor progression and metastasis [6,7]. Importantly, these properties are not intrinsic to CSCs alone but are dynamically regulated by the tumor microenvironment (TME) and the specialized niches that support them [8].

Accurately modeling cancer heterogeneity is essential for the development of novel therapeutic strategies and the advancement of personalized medicine. In response to this need, three-dimensional (3D) organoid technology is garnering increasing interest [9]. 3D organoids better replicate the structural complexity and cellular interactions of tumors compared to traditional two-dimensional (2D) culture systems. Organoid models provide a useful platform to preserve intra-tumoral heterogeneity by maintaining multiple co-existing cell populations derived from the original tumor [10]. This enables the investigation of clonal dynamics and cell–cell interactions that are difficult to capture in conventional models [11]. However, current cancer organoid models still face significant limitations, including the incomplete incorporation of key components of the TME, such as immune and vascular elements. Additionally, organoid establishment requires extended periods, and long-term culture can lead to genetic alterations [10,12].

To overcome these challenges and more precisely recapitulate the complexity of the tumor ecosystem, patient-derived organoids (PDOs) have emerged as a promising model. PDOs are capable of reproducing the architecture and cellular diversity of native tumors, thereby playing a critical role in predicting therapeutic responses and unraveling tumor heterogeneity [11]. Beyond structural recapitulation, PDOs retain patient-specific genomic and phenotypic features [13]. This enables more accurate modeling of inter- and intra-tumoral heterogeneity [14].

However, despite these advantages, organoid-based models still face limitations in accurately predicting therapeutic responses. To address these challenges, efforts to integrate AI into organoid research are gaining increasing momentum. AI-driven approaches enable sophisticated analysis of organoid datasets and improve the prediction of drug responsiveness, thus accelerating progress in precision oncology [15]. This further enhances the translational relevance of organoid-based models in predicting patient-specific therapeutic responses [16]. Such integrative approaches are expected to further improve the clinical applicability of organoid-based systems by strengthening their predictive performance in therapeutic response assessment [17].

In this review, we discuss the crosstalk between CSCs and the TME, explore the mechanisms driving spatial and cellular heterogeneity in cancer, and examine the application and limitations of CSC-derived 3D organoid models. We further highlight how the convergence of organoid and AI technologies is shaping future strategies for personalized cancer therapy.

## 2. Crosstalk Between Cancer Stem Cells and the TME

### 2.1. Features of CSCs

Although CSCs constitute only approximately 0.05–3% of the entire cancer cell population, they are recognized as key drivers of tumor initiation, metastasis, and intratumoral heterogeneity [18,19]. Although CSCs share many characteristics with normal stem cells, two key differences distinguish the two cell types. First, signaling pathways that regulate survival, proliferation, self-renewal, and differentiation are aberrantly activated or suppressed in CSCs, promoting tumor initiation and progression, whereas in normal stem cells, these pathways are tightly regulated [20]. Second, normal stem cells typically remain dormant during adulthood until regenerative needs arise, whereas CSCs maintain regenerative capacity and remain persistently active [21].

A defining feature of CSCs is their ability to adapt to environmental stress. CSCs are capable of self-renewal even under hypoxic and acidic conditions, proliferating indefinitely and differentiating into various malignant cell types. These traits enable CSCs to survive after therapy and persist within the host, serving as a major cause of tumor recurrence [19,22,23]. Additionally, they can enter a dormant state in response to environmental stresses such as DNA damage, molecular stress, and therapy-induced stress, conferring a higher level of therapy resistance compared to regular cancer cells. Consequently, they are strongly resistant to both radiotherapy and chemotherapy [24]. Furthermore, they interact with immune cells to create an immunosuppressive TME, which in turn facilitates tumor initiation and progression [25].

These intrinsic properties of CSCs are closely influenced by external microenvironmental cues. Therefore, these features underscore the intricate crosstalk between CSCs and the TME, wherein reciprocal interactions not only reinforce stemness properties but also contribute significantly to the development of therapeutic resistance [26].

### 2.2. Signaling Pathways That Regulate CSCs

**Figure 1:**
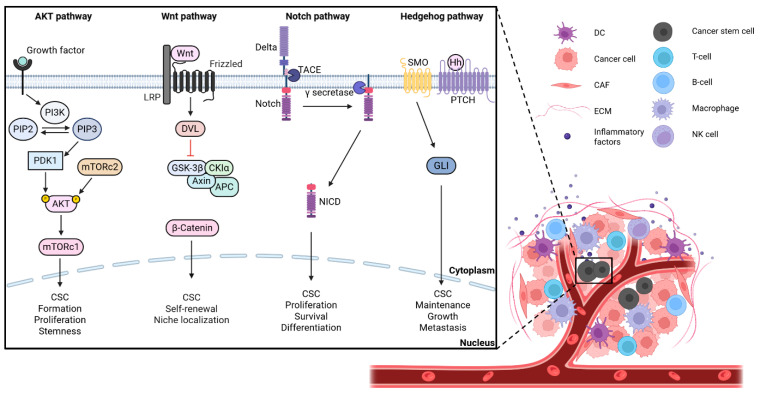
Regulation of cancer stem cells by the tumor microenvironment and key signaling pathways. The tumor microenvironment comprises cancer stem cells (CSCs), dendritic cells (DCs), cancer cells, T cells, B cells, macrophages, natural killer (NK) cells, extracellular matrix (ECM), cancer-associated fibroblasts (CAFs), and inflammatory factors. CSCs within the tumor microenvironment are regulated by key signaling pathways, including AKT, Wnt, Notch, and Hedgehog pathways. Created with BioRender.com.

CSCs play a central role in tumor initiation, maintenance, metastasis, and the acquisition of therapeutic resistance through interactions with numerous signaling pathways (Figure 1).

#### 2.2.1. PI3K/AKT/mTOR Signaling Pathway

The PI3K/AKT/mTOR signaling pathway is one of the most critical regulators of CSC formation, proliferation, and stemness. Aberrant activation promotes CSC expansion, whereas inhibition suppresses CSC growth, induces apoptosis, and reduces metastatic potential while enhancing radiosensitivity [27]. At the molecular level, PI3Ks generate PIP3, leading to sequential activation of PDK1, AKT, and mTOR complexes, which regulate cellular growth, metabolism, and protein synthesis [28,29,30,31,32]. Given its broad role in angiogenesis and differentiation, the PI3K/AKT/mTOR axis is a key modulator of CSC behavior across multiple cancers [33].

#### 2.2.2. Wnt/β-Catenin Signaling Pathway

The Wnt/β-catenin pathway regulates CSC self-renewal, proliferation, and resistance to therapy, and its dysregulation promotes CSC characteristics and tumor progression [34,35]. Mechanistically, Wnt signaling inhibits β-catenin degradation, allowing its nuclear translocation and activation of target genes including MYC, cyclin D1, and SOX2 [36]. This pathway plays a central role in tumorigenesis and CSC maintenance [37].

#### 2.2.3. Notch Signaling Pathway

The Notch signaling pathway is a key regulator of CSC self-renewal, survival, and differentiation, and contributes to chemoresistance [38]. Activation occurs through ligand-receptor interaction followed by cleavage events that release the Notch intracellular domain, which regulates transcription of target genes such as HES1 and HEY1 [39]. Targeting Notch signaling can modulate CSC proliferation and tumor progression, highlighting its therapeutic potential [40].

#### 2.2.4. Hedgehog (Hh) Signaling Pathway

The Hedgehog signaling pathway plays a critical role in CSC maintenance, stemness, and metastatic potential and is associated with chemoresistance [21,40]. Hh ligands bind to PTCH receptors, relieving inhibition of Smoothened and leading to activation of Gli transcription factors that regulate target genes such as c-Myc and cyclin D1 [41,42].

Collectively, these signaling pathways integrate intrinsic and extrinsic cues to regulate CSC properties and contribute to the generation of intratumoral heterogeneity [7]. Through regulation of self-renewal, differentiation, and plasticity, they enable CSCs to generate diverse cellular states in response to dynamic spatial and temporal microenvironmental cues. Thus, dysregulated signaling in CSCs establishes a functional link between signaling pathway activity and intratumoral heterogeneity, contributing to therapeutic resistance and tumor progression [43]. Such signaling dynamics can be recapitulated and systematically investigated in organoid-based systems, providing physiologically relevant platforms for studying CSC-driven heterogeneity [44].

### 2.3. Niche and TME of CSCs

Niche refers to a specialized microenvironment that supports CSC survival, self-renewal, and tumor progression [45,46]. CSCs establish and remodel their niche within the TME, thereby enhancing their resistance to therapy and promoting tumor growth [45]. The TME plays a critical role in regulating tumor progression, metastasis, therapeutic response, and heterogeneity, primarily through complex cellular interactions and immunosuppressive mechanisms [47,48,49]. CSC interactions with TME components are key drivers of tumor recurrence, metastasis, and drug resistance, highlighting the importance of their dynamic crosstalk [50].

The TME consists of diverse cellular and non-cellular components, including immune cells, stromal cells, extracellular matrix (ECM), and soluble factors, which collectively influence tumor behavior and CSC function [51,52]. In particular, inflammatory cytokines such as IL-1, IL-6, and IL-8 mediate CSC–TME interactions and contribute to tumor progression and therapeutic resistance [53]. Structural components, including CAFs and ECM, play essential roles in niche formation, extracellular remodeling, and mechanical signaling, thereby promoting CSC maintenance and metastasis [54,55].

As a key component of the TME, ECM-mediated mechanical properties also regulate CSC differentiation potential [56]. In various cancers, cancer-associated adipocytes form an extensive ECM and actively contribute to cancer progression through mechanisms such as inducing senescence-like phenotypes and regulating immune responses [57,58]. CSCs promote angiogenesis through interactions with endothelial cells and by producing angiogenic factors, thereby supporting tumor growth [59].

Signaling pathways such as PI3K/AKT/mTOR, Wnt/β-catenin, Notch, and Hedgehog mediate CSC–TME interactions and regulate CSC self-renewal, differentiation, and survival, ultimately promoting therapeutic resistance and metastasis.

## 3. Mechanisms Driving Spatial and Cellular Heterogeneity in Cancer

### 3.1. Cancer Heterogeneity

Intratumor heterogeneity refers to the coexistence of genetically and phenotypically distinct subpopulations of cancer cells within a single tumor mass. It contributes to tumor progression, metastasis, and therapeutic resistance and is widely recognized as a major driver of tumor evolution and treatment failure across multiple cancer types [4,5]. Depending on its extent, it may also facilitate immune evasion by enabling tumors to suppress anti-tumor immune responses, thereby contributing to adverse clinical outcomes [60]. Despite its clinical significance, the timing of its emergence, the mechanisms underlying its maintenance, and its functional implications during tumor progression remain incompletely understood [61]. Recent advances in single-cell sequencing and spatial transcriptomics have enabled high-resolution characterization of tumor heterogeneity, providing insights into cellular diversity and microenvironmental interactions [62,63,64,65].

In recent years, intratumor heterogeneity has been increasingly recognized as a dynamic evolutionary process driven by the interplay between genetic diversification and selective pressures imposed by the tumor microenvironment [66]. These selective pressures promote phenotypic plasticity, thereby enabling cancer cells to transition between distinct cellular states and enhancing their adaptability under therapeutic and environmental stress [67]. In addition, non-genetic heterogeneity arising from epigenetic regulation and transcriptional variability further contributes to functional diversification among tumor cell populations [68]. Furthermore, hierarchical organization within tumors, often driven by CSC populations, supports the continuous generation of phenotypically diverse progeny cells, thereby reinforcing intratumoral heterogeneity [7]. Consequently, both intrinsic genetic alterations and extrinsic microenvironmental factors collectively shape tumor heterogeneity, ultimately driving the emergence of diverse subclonal populations [7].

These heterogeneous cellular states exhibit differential therapeutic sensitivities, leading to selective survival of resistant subclones and ultimately contributing to treatment failure [69]. Intratumor heterogeneity can be broadly categorized into spatial and cellular heterogeneity. Intratumor spatial heterogeneity arises from localized differences in microenvironmental conditions within the tumor, including variations in oxygen availability, nutrient gradients, and stromal interactions [70]. In parallel, cellular heterogeneity reflects variations among individual cancer cells driven by genetic, epigenetic, and transcriptional differences, leading to diverse phenotypic states within the tumor [71]. Together, these interconnected layers of heterogeneity shape tumor behavior and therapeutic response, highlighting the need for precise strategies to analyze and target tumor diversity.

### 3.2. Mechanisms Driving Cancer Heterogeneity

This section provides a summary of six principal mechanisms that drive cancer heterogeneity (Table 1).

The tumor microenvironment (TME) is a major driver of intratumoral heterogeneity because dynamic interactions among stromal, immune, and vascular components continuously reshape tumor cell states [1,72,73]. By promoting tumor cell plasticity, the TME influences tumor progression, clinical outcomes, and spatial–temporal heterogeneity. TME-derived stress conditions, such as hypoxia and immune pressure, actively reshape clonal dynamics and promote adaptive phenotypic transitions [52].

Extrachromosomal DNA (ecDNA) is now recognized as a major source of genetic heterogeneity in cancer because it enables high-level oncogene amplification and atypical inheritance during mitosis [74,75]. These properties facilitate rapid diversification of tumor cell populations and contribute to therapeutic resistance by enabling dynamic copy number variation and uneven segregation under selective pressure [76].

Therapeutic interventions such as chemotherapy and immunotherapy impose selective pressure on tumor cells, favoring the survival and expansion of resistant clones [77]. As tumors evolve, genetically and epigenetically distinct subclones accumulate additional alterations, giving rise to populations with increased invasiveness and treatment resistance [78]. This process results in branched evolutionary trajectories, where distinct subclones adapt differently to therapeutic and environmental pressures [79].

Cell–cell fusion contributes to tumor heterogeneity by generating hybrid cells with altered phenotypes and functional properties [80,81]. However, its effects can be context-dependent, reflecting both tumor-promoting and potentially suppressive consequences. Thus, cell fusion should be viewed as a contributory rather than universally dominant mechanism of heterogeneity [82].

Chromosomal instability (CIN) promotes intratumoral heterogeneity by continuously generating diverse aneuploid states [81,83]. Whole-genome doubling (WGD) further potentiates this process by buffering genomic loss and enabling continued genomic instability, thereby providing a substrate for selection and tumor adaptation [83,84].

CSCs contribute substantially to tumor heterogeneity through their intrinsic plasticity, which enables dynamic transitions in phenotype and function [7,85,86,87]. In addition, interactions between CSCs and their niche further amplify tumor diversity [21]. CSC plasticity allows bidirectional transitions between stem-like and differentiated states, contributing to dynamic intratumoral heterogeneity [43].

Collectively, these mechanisms interact rather than act independently, forming a dynamic network that continuously drives tumor heterogeneity.

### 3.3. Spatial and Cellular Heterogeneity

#### 3.3.1. Spatial Heterogeneity

Spatial heterogeneity arises from region-specific differences in oxygen availability, nutrient gradients, stromal composition, and immune contexture, which collectively generate localized selective pressures within tumors [88]. These spatially distinct cellular states are further reinforced by genomic instability, transcriptional plasticity, and dynamic interactions with the surrounding microenvironment [89]. Spatial heterogeneity critically influences tumor evolution and therapeutic response by creating region-specific differences in cell state, signaling activity, and metabolic adaptation [90].

**Figure 2:**
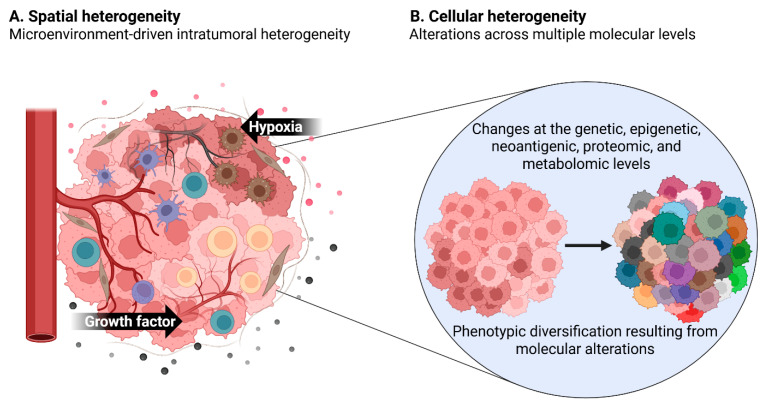
Spatial and cellular heterogeneity. (**A**) Spatial heterogeneity is derived from microenvironmental differences occurring in different areas of the same tumor, and these local differences influence tumor progression and treatment responsiveness. (**B**) Cellular heterogeneity results from changes at the genetic, epigenetic, neoantigenic, proteomic, and metabolomic levels, leading to phenotypic diversification at the single-cell level in tumors. Created with BioRender.com.

In a broader sense, spatial heterogeneity may also encompass differences between primary and metastatic lesions [2,91]. However, from a fundamental perspective, it refers to localized variations in the accessibility and distribution of nutrients and growth factors within the tumor, driven by complex interactions among cancer cells, immune cells, stromal cells, vascular structures, and microenvironmental factors such as hypoxia [92] (Figure 2A). Moreover, analysis of region-specific genetic discrepancies can provide insights into clonal divergence and help estimate the timing of metastatic events during disease progression [2]. Recent advances in spatially resolved deep-learning algorithms, combined with the integration of spatial transcriptomics (ST) and CODEX data, have enabled high-resolution characterization of region-specific transcriptional programs and tissue architecture, facilitating the identification of spatial subclones, immune niches, and microenvironmental heterogeneity within tumors [90]. The spatial organization of cancer cells, stromal populations, and immune niches strongly influences disease progression and therapeutic response, and these interactions can now be profiled at high resolution using spatial transcriptomics and multiplex imaging approaches [70]. Notably, spatial intratumor heterogeneity affects both therapeutic responsiveness and durability, making its accurate characterization a crucial foundation for the development of effective treatment strategies [93].

Recent spatial profiling studies have shown that intratumoral spatial organization is closely associated with clinical outcome and therapeutic resistance across multiple tumor types [94,95,96,97,98,99,100,101,102,103]. Together, these findings demonstrate that spatial heterogeneity directly shapes region-specific tumor behavior, enabling more precise analysis of therapeutic response and resistance; however, accurately resolving this complexity remains a major challenge. Emerging evidence suggests that CSC subpopulations contribute to spatial heterogeneity by adapting to distinct microenvironmental niches and promoting region-specific clonal expansion. These region-specific adaptations may also contribute to metabolic heterogeneity within the tumor microenvironment.

#### 3.3.2. Cellular Heterogeneity

Cellular heterogeneity arises from genetic diversity and dynamic molecular interactions that generate distinct transcriptional, epigenetic, and functional states among individual tumor cells (Figure 2B).

These distinct cellular states are highly dynamic and can reversibly transition in response to microenvironmental signals and therapeutic pressures, thereby contributing to tumor progression, metastasis, and treatment resistance [104]. This heterogeneity becomes more readily observable when it influences phenotypic characteristics such as cell survival time, proliferation rate, and size distribution, with genetic mutations recognized as a primary driving force [105]. These molecular alterations collectively drive phenotypic diversification, resulting in the emergence of functionally distinct tumor cell populations within the same tumor [106]. CSC subpopulations have been suggested to contribute to clonal diversification and the maintenance of heterogeneous tumor cell states.

Cellular heterogeneity is now most clearly resolved using single-cell RNA sequencing, which enables high-resolution characterization of transcriptional diversity, rare cellular subpopulations, and dynamic functional states within tumors [64,107,108,109,110]. Furthermore, integration of single-cell transcriptomic data with trajectory inference and multimodal analyses enables the reconstruction of cellular hierarchies and dynamic state transitions within tumor ecosystems [111]. More recently, single-cell ATAC-seq and related epigenomic profiling approaches provide additional insights into chromatin accessibility and regulatory heterogeneity, thereby capturing epigenetic mechanisms underlying diverse cellular states [112,113]. Accordingly, single-cell level heterogeneity remains a major challenge for cancer diagnosis and treatment [114]. In parallel, lineage tracing approaches have provided direct insight into clonal evolution by reconstructing phylogenetic relationships and tracking dynamic cell-state transitions during tumor progression and metastasis [115,116,117]. These advanced technologies can be integrated with organoid-based models to functionally recapitulate and dissect CSC-driven heterogeneity in controlled experimental settings.

Cellular heterogeneity is a fundamental feature observed across diverse cancer types, driven by both intrinsic genetic alterations and microenvironmental influences [118,119,120,121,122]. Collectively, these findings demonstrate that cellular heterogeneity is not merely associated with cellular plasticity but actively drives tumor adaptation and therapeutic resistance.

## 4. 3D Organoid Technology and Its Application in Cancer Research

### 4.1. Introduction to 3D Organoid Technology

Patient-derived xenografts (PDXs) and genetically engineered mouse models, as representative preclinical cancer models, recapitulate the 3D architecture and TME. However, their clinical success rate is approximately only 3%, primarily due to ethical concerns, technical limitations, and insufficient clinical predictability [123]. To overcome these limitations, interest in 3D organoid-based technology has increased.

**Figure 3:**
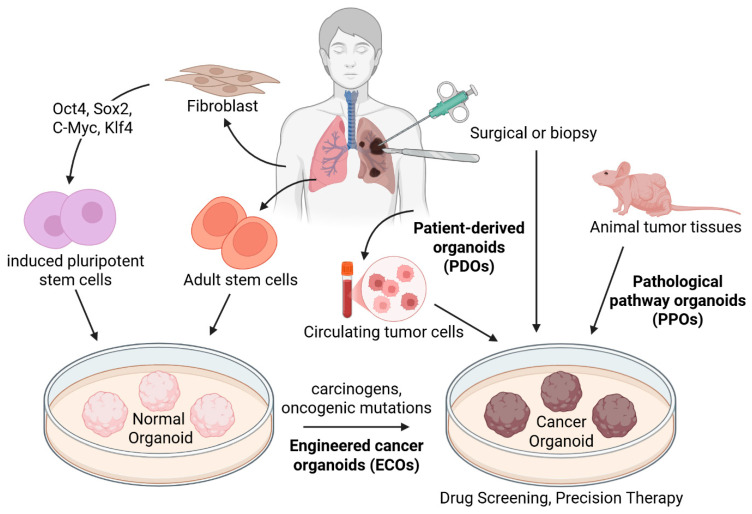
Sources and generation pathways of cancer organoids. Normal organoids are generated from induced pluripotent stem cells (iPSCs) or adult stem cells (ASCs). Cancer organoids are categorized into engineered cancer organoids (ECOs), patient-derived organoids (PDOs), and pathological pathway organoids (PPOs). ECOs are generated by treating normal organoids with carcinogens or by oncogenic mutations. PDOs are derived from circulating tumor cells or surgical and biopsy specimens of patient tumors. PPOs are established tumor tissues from animal models. Created with BioRender.com.

3D organoids are miniature structures that can be generated from pluripotent or adult stem cells, as well as from patient-derived tumor tissues, and are capable of recapitulating tissue architecture, cellular function, and signaling pathways [124,125,126] (Figure 3).

3D organoid technology is both histologically and genetically similar to tumor tissue, thereby bridging the gap between 2D cell culture and mouse models. Furthermore, the ability to maintain long-term culture and cryopreserve organoids facilitates various applications [127].

### 4.2. Applications of Organoids in Cancer

PDX models have several limitations, including a low success rate, limited throughput, low screening efficiency, high costs, and prolonged duration. For these reasons, PDO models have gained attention as a more efficient platform for drug evaluation [128,129]. PDOs can be generated from tumor tissues collected at various stages of cancer progression, including the primary tumors, circulating tumor cells, and secondary metastatic lesions [123]. PDOs can be established through various approaches, including CSC isolation, CTC-derived organoid generation, and dissociation of tumor tissues [125,130,131,132,133]. These PDOs are used for individual drug response assessment through drug screening. A recent study demonstrated that PDOs can serve as functional biomarkers for predicting treatment response [134]. This highlights their ability to capture patient-specific differences in treatment response.

## 5. Overcoming Technical and Biological Barriers in CSC-Derived Organoid Models

Table 2 summarizes key technical and biological obstacles associated with CSC-derived organoid models, along with proposed strategies to overcome each.

### 5.1. Rarity of CSCs and Difficulty Isolating Them

CSCs constitute only a minor fraction of tumor cells, making their isolation inherently difficult and limiting the efficiency of CSC-derived organoid establishment [19]. Common enrichment approaches rely on CSC-associated surface markers, such as CD24, CD44, CD133, and EpCAM, in combination with FACS, MACS, or flow cytometry [135,136,137,138,139,140,141,142,143,144]. Organoid generation generally requires mechanical dissociation followed by enzymatic digestion to obtain single-cell suspensions [168]. Careful optimization of tissue-processing conditions is therefore essential to minimize cellular damage and improve CSC recovery [145,146]. However, this limitation is not solely technical, because marker-based isolation does not necessarily capture the full biological diversity of CSC states. CSC populations are increasingly understood as dynamic and plastic rather than fixed entities, meaning that static marker panels may preferentially enrich only selected subpopulations [18,169]. Thus, the key challenge lies not only in improving CSC yield, but also in ensuring that isolated cells faithfully represent intratumoral heterogeneity.

### 5.2. Lack of Standardized Culture Conditions for CSCs and Cancer Organoids

Culture conditions for CSCs remain poorly standardized, leading to variability across studies and limiting reproducibility [170]. Similarly, cancer organoid systems exhibit substantial variability due to differences in cytokine composition and TME-related factors, which directly affect organoid formation efficiency [171]. Achieving reproducibility is critical for clinical translation; however, standardized protocols remain difficult to implement across laboratories. Because many cancer organoid systems rely on CSC populations, understanding CSC-specific niche requirements and regulatory factors is essential for improving model consistency [147]. However, complete standardization may be difficult to achieve, as organoid culture conditions often need to be optimized according to tumor type, tissue origin, and biological context [148]. Therefore, the central challenge is not simply to impose uniform protocols, but to establish standardized frameworks that preserve reproducibility while accommodating biological diversity [147].

### 5.3. Lack of TME Components Such as Immune Cells and Vasculature

Current CSC-derived organoid models often lack key components of the TME, including immune cells, vascular structures, fibroblasts, and extracellular matrix interactions [149,150]. As a result, these models have limited capacity to recapitulate complex cell–cell and cell–matrix interactions observed in vivo. To address these limitations, advanced approaches such as co-culture systems incorporating immune and stromal cells, as well as microengineered organoid-on-chip platforms, have been developed to better recapitulate the TME [149,150,151,152,153,154,155,156]. These platforms have shown promise in improving physiological relevance and enabling the investigation of TME interactions under more realistic conditions [129]. However, increasing model complexity may introduce additional experimental variability, which can complicate reproducibility across different systems and laboratories. Current models cannot fully reproduce the spatial organization and cell–cell interactions observed in vivo, limiting their ability to reflect tumor heterogeneity.

### 5.4. Limited Predictive Accuracy of CSC-Derived Organoids

Cancer organoids provide improved modeling of tumor–immune interactions compared to conventional 2D cell lines and some animal models, supporting their use in personalized treatment prediction [172]. However, cancer organoid-based treatment response models still face technical limitations, and such limitations have also been noted in CSC-derived cancer organoids.

CSC-derived PDOs have demonstrated promising predictive performance in certain clinical contexts, such as radiotherapy response, yet their outputs do not fully recapitulate actual patient outcomes [134]. A study using CRC PDOs reported a treatment response prediction accuracy of approximately 76%, indicating that there are still limitations in precisely predicting personalized treatment outcomes [173]. To address these limitations, the concept of digital organoids has been introduced, integrating organoid-derived drug response data with deep learning-based analytical frameworks [157]. For instance, 3D live imaging platforms such as BEHAV3D enable dynamic analysis of cell–cell interactions and TME changes within organoids, providing additional layers of data for response prediction [10,158]. However, improvements in computational modeling cannot fully compensate for limitations in the biological fidelity of organoid systems. In particular, incomplete representation of TME components and tumor heterogeneity may constrain the reliability of AI-driven predictions. Therefore, enhancing predictive accuracy requires not only advances in computational approaches but also improvements in the biological complexity and representativeness of organoid models.

### 5.5. Limitations of CSC-Derived Organoids in Reproducing Tumor Heterogeneity

Organoids derived from circulating tumor cells or CSCs isolated from a single tumor region are usually generated from only one or a few clones. Consequently, they cannot fully capture the broad heterogeneity seen in real tumors [147]. To overcome this limitation, strategies such as clonal organoid systems incorporating CSCs from multiple regions, as well as multifocal organoids derived from spatially distinct tumor sites, have been proposed [159,160,161].

In addition, AI-based approaches have emerged as complementary tools for analyzing and characterizing tumor heterogeneity in organoid systems. AI-based deep learning models enable precise analysis of key characteristics such as organoid viability [162], differentiation tendencies [163], and drug responses [164], and models utilizing PDOs to characterize tumor heterogeneity are also being developed [165]. For instance, image-based deep learning has been used to identify morphological heterogeneity in CRC organoids [166]. In another study, oral cancer PDOs were classified into three morphological subtypes: normal-like, dense, and grape-like using AI-based scoring methods. Importantly, these subtypes were not only visually distinct but also showed meaningful differences in patient prognosis [167]. However, AI-based approaches primarily enhance data interpretation rather than fundamentally improving the biological representativeness of organoid models. In particular, organoids derived from limited clonal populations may still fail to capture spatial and cellular heterogeneity, which cannot be fully resolved through computational analysis alone. Therefore, improving heterogeneity modeling requires not only advanced analytical tools but also integrative strategies that enhance the biological representativeness and diversity of organoid systems.

## 6. Therapeutic Perspectives: Targeting Heterogeneity Using 3D Cancer Organoid Models

**Figure 4:**
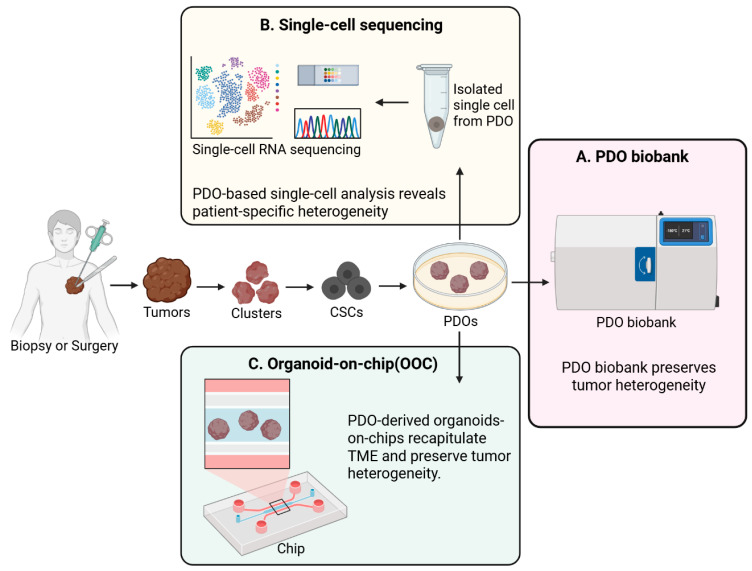
Patient-derived organoid (PDO)-based strategies targeting tumor heterogeneity. Tumor tissues obtained via biopsy or surgery are used to generate PDOs from clusters of cancer stem cells (CSCs). (**A**) PDO biobanks preserve tumor heterogeneity and enable long-term storage. (**B**) Single-cell RNA sequencing of PDOs reveals patient-specific and intratumoral heterogeneity. (**C**) Organoid-on-chip (OOC) systems recapitulate the tumor microenvironment and support functional assessment of heterogeneity-related phenotypes. Created with BioRender.com.

Recent technological advancements have enabled the development of multiple PDO-based strategies to address tumor heterogeneity (Figure 4).

### 6.1. PDO Biobanks

Traditional cancer biobanks typically store normal tissues, tumor tissues, and blood samples. Unlike conventional specimen repositories, PDO biobanks function as renewable living resources because organoids can be cryopreserved, revived after thawing, and expanded for repeated molecular and pharmacologic analyses [174]. This renewable nature is particularly advantageous for longitudinal studies, assay validation, and cross-platform comparisons that require the same patient-derived material to be tested multiple times under standardized conditions [175]. Importantly, recent studies have shown that PDOs can also be generated from clinically accessible specimens such as needle biopsies, ascites, and pleural effusions, thereby broadening the applicability of organoid biobanking beyond surgically resected tumors [176]. Such flexibility is highly relevant for advanced or inoperable cancers, in which repeated tissue acquisition is difficult but functional testing remains clinically important [177].

Biobanks utilizing PDOs derived from various cancer types reproduce the pathological, genomic, proteomic, and morphological characteristics of their parental tumors [133]. Beyond preserving histopathologic and genomic features, contemporary PDO biobanks increasingly serve as integrative platforms that link multi-omics profiles with therapeutic vulnerabilities across molecular subtypes [178]. Notably, they also enable the capture of intratumoral heterogeneity at the single-cell level. These characteristics highlight the utility of PDO-derived biobanks as platforms for high-throughput drug screening and evaluation of therapeutic responses while maintaining the heterogeneity of parental tumors [179]. To more faithfully preserve intra-tumoral heterogeneity, recent organoid biobanking strategies have incorporated multi-region sampling from spatially distinct tumor areas rather than relying on a single specimen from each patient [178]. Accordingly, PDO biobanks should be viewed not merely as repositories of expandable cultures, but as structured resources for functionally interrogating spatial and clonal heterogeneity [180].

PDO biobanks also demonstrate strong clinical scalability and applicability, such as large-scale disease modeling and new drug development. Recently, a PDO biobank established from patients with bladder cancer was found to accurately reflect tumor heterogeneity [180]. Such findings are important because they indicate that organoid biobanks can preserve not only inter-patient diversity but also clinically meaningful variation in treatment sensitivity across disease stages and molecular subgroups [129]. Additionally, PDO-derived biobanks have been established for various cancer types, including prostate, colon, pancreatic, gastric, renal, breast, ovarian, and bladder cancers. These biobank datasets are continuously utilized to study tumor heterogeneity and to develop precision treatment strategies [181]. As these collections expand, they enable cohort-level analyses of resistance mechanisms, rare molecular subtypes, and cross-patient response patterns that are difficult to resolve using individual patient samples alone [182].

Thus, PDO biobanks can play a pivotal role as precision medicine-based platforms for patient-tailored treatment strategies while precisely preserving the biological characteristics of tumor heterogeneity.

### 6.2. Single-Cell Sequencing

Bulk sequencing has inherent limitations in accurately analyzing intratumoral heterogeneity because it provides average genetic information aggregated from heterogeneous cell populations. However, the advent of single-cell sequencing has helped overcome this limitation, making it possible to analyze both spatial and cellular heterogeneity, which could not be accurately assessed using bulk sequencing. Importantly, the integration of single-cell RNA sequencing with PDO models enables the identification of diverse cellular states and lineage hierarchies within tumors, which are often masked in bulk-level analyses [183]. Specifically, single-cell sequencing enables precise analysis of key elements of tumor heterogeneity, including clonal heterogeneity, cellular crosstalk, CSCs, tumor metastasis, CTCs, treatment resistance, spatial organization, and interaction mechanisms within the TME [7]. Recently, single-cell RNA sequencing has been integrated with PDOs in novel approaches aimed at analyzing personalized cancer heterogeneity and developing individualized treatment strategies. Furthermore, coupling single-cell transcriptomic profiling with drug perturbation assays in PDOs allows the identification of resistant subpopulations and the prediction of patient-specific therapeutic responses [184]. Generally, PDOs are dissociated into single cells before single-cell RNA sequencing is performed. This process not only enables the identification of heterogeneity between patients but also confirms that PDOs reflect the gene expression patterns of parental tumors and maintain their heterogeneity over time [63]. In a study using breast cancer PDOs, single-cell sequencing was performed after dissociating the PDOs into single cells. The results showed that each PDO retained the heterogeneity of its parental tumor while also exhibiting intra-organoid heterogeneity [185]. More recently, studies have combined single-cell sequencing with spatial transcriptomics to reconstruct tumor architecture and microenvironmental interactions at high resolution [186]. Future approaches integrating single-cell multi-omics and machine learning are expected to further enhance the predictive power of PDO-based platforms in precision oncology [187].

### 6.3. Organoid-on-Chip (OOC) Technologies

Organoid-on-a-chip (OOC) is a system that combines organoids with organ-on-a-chip technology and has attracted considerable attention in the field of 3D cancer modeling. Traditional cancer organoids have limitations in fully reproducing the TME [188]. OOC platforms integrate organoid cultures with microfluidic systems, enabling more precise control of biochemical gradients, fluid flow, and cell–cell interactions than conventional static organoid culture [189]. These limitations largely stem from the insufficient representation of dynamic microenvironmental cues, including perfusion, stromal interactions, and vascular components, as well as the CSC niche, in conventional organoid systems [190]. OOC systems have been proposed to address this limitation. OOC has been reported to precisely recapitulate the TME [191]. Furthermore, OOC systems composed of patient-derived cells or tissues can accurately analyze the drug resistance mechanisms in tumor cells and enable drug sensitivity assessment within a shorter time frame (typically within 14 days) compared to traditional cancer organoids [192]. Particularly, RNA-seq-based principal component analysis demonstrated that patient-specific heterogeneity was preserved in CRC OOC models [193]. Recent studies have also begun to integrate multi-omics strategies with OOC platforms to achieve a higher-resolution characterization of inter- and intra-patient heterogeneity [194,195]. Additionally, vascularized chip-based organoid models are being evaluated as systems that can effectively capture heterogeneity resulting from cancer angiogenesis and metastasis [196]. These vascularized models allow real-time monitoring of tumor–vasculature interactions and provide a tractable platform for studying angiogenesis and metastatic dissemination [197]. Such platforms can be integrated not only with cancer organoids but also with organoids derived from various organs, such as the retina and brain, and can be broadly utilized to investigate tissue-specific heterogeneity and to develop precision therapeutic strategies [198,199]. In addition, integration with AI and advanced image analysis is expected to further improve the predictive and analytical capabilities of OOC systems [200].

OOC systems enable controlled modeling of tumor–microenvironment interactions, which are difficult to capture in conventional organoid cultures. This allows precise manipulation of biochemical and physical cues, enabling real-time analysis of tumor behavior under defined microenvironmental conditions.

## 7. Conclusions

This review summarizes recent advances demonstrating that CSC-derived organoids contribute to the establishment and maintenance of the core features of tumor heterogeneity. It also explores the potential of patient-derived CSC organoids as next-generation platforms for accurately recapitulating tumor heterogeneity.

CSCs play a central role in the formation and maintenance of tumor heterogeneity. The self-renewal and differentiation abilities of CSCs, along with their interactions with the surrounding niche, are major drivers of clonal diversity and treatment resistance. CSC-based organoid models are attracting attention as a novel and effective approach to model these defining characteristics of CSCs. CSC-derived organoids provide robust platforms for accurately modeling intratumoral heterogeneity and the complex interactions within the TME, which are challenging to reproduce using conventional 2D culture systems or animal models. Organoids derived from patient-derived CSCs preserve the structural and functional characteristics of primary tumors, highlighting their clinical potential for predicting therapeutic responses and patient prognosis.

Technologies such as organoid co-culture, PDO biobanking, single-cell RNA sequencing, and OOC platforms have further improved organoid models by enabling more accurate reproduction of tumor heterogeneity, higher biological precision, and better prediction of treatment responses. Organoid-based platforms are anticipated to enable the tracking of tumor heterogeneity dynamics, identification of patient-specific therapeutic targets, and prediction of treatment responses, highlighting their potential to improve the interpretation of patient-specific treatment responses.

## Figures and Tables

**Table 1:** Molecular and cellular determinants of tumor heterogeneity.

Mechanism	Function
TME	The TME contributes to spatial and temporal heterogeneity within tumors and significantly influences prognosis and therapeutic response [1,72,73].
ecDNA	ecDNA serves as a major carrier of amplified oncogenes, promoting tumor progression and heterogeneity by enabling certain cancer cells to harbor numerous oncogenic drivers that contribute to intratumoral diversity and drug resistance [74,75,76].
Clonal evolution	As cancer progresses, cells accumulate genetic and epigenetic alterations, giving rise to new clones with distinct characteristics such as therapy resistance and invasiveness, which drive tumor heterogeneity [77,78,79].
Cell–cell fusion	Tumor cells can fuse with other cell types, generating hybrid cells that contribute to increased tumor heterogeneity and phenotypic variability [80,81,82].
CIN	CIN leads to the persistent generation of heterogeneous aneuploid states, thereby enhancing tumor heterogeneity [83,84].
CSCs	CSCs possess phenotypic and functional plasticity, which contributes to intratumoral heterogeneity by giving rise to diverse cellular subpopulations. Both CSCs and their supportive niches exacerbate this heterogeneity within the tumor [7,85,86,87].

Abbreviations: TME, tumor microenvironment; ecDNA, extrachromosomal DNA; CIN, chromosomal instability; CSCs, cancer stem cells.

**Table 2:** Technical and biological limitations of CSC-derived organoid models and strategies for improvement.

Limitation	Description	Proposed Strategy
Rarity of CSCs and difficulty isolating them	CSCs make up only 0.05–3% of tumor cells [19], making their isolation and organoid establishment technically challenging.	1. Use CSC surface markers (e.g., CD24, CD44, CD133, EpCAM) and apply FACS, MACS, or FCM [135,136,137,138,139,140,141,142,143,144] 2. Optimize enzymatic digestion to improve CSC viability [145,146].
Lack of standardized culture conditions	CSC and cancer organoid culture conditions, including cytokines and TME factors, are not standardized, causing variability [147,148].	Standardize protocols based on CSC niche and self-renewal factors [147,148].
Absence of TME components	Current models lack immune cells, fibroblasts, vascular endothelial cells, and ECM, limiting tumor-like cellular interactions [149,150].	1. Apply co-culture systems with T cells, TAMs and endothelial cells [149,150,151,152,153,154,155,156]. 2. Use organoid-on-chip technologies [149,150,151,152,153,154,155,156].
Inaccurate treatment prediction	CSC-derived PDOs show promising treatment predictability, but fail to fully match clinical outcomes [134].	1. Develop digital organoids integrated with deep learning [157]. 2. Utilize 3D imaging-based platforms (e.g., BEHAV3D) [10,158].
Limited modeling of tumor heterogeneity	Organoids derived from a single tumor region or a limited number of clones are unable to fully recapitulate the genetic and phenotypic diversity observed within tumors [147].	1. Employ clonal organoid systems [159,160,161]. 2. Generate multifocal organoids from diverse tumor sites [159,160,161]. 3. Apply AI-based methods to identify and classify tumor heterogeneity [162,163,164,165,166,167]

Abbreviations: CSCs, cancer stem cells; FACS, fluorescence-activated cell sorting; MACS, magnetic activated cell sorting; FCM, flow cytometry; TME, tumor microenvironment; ECM, extracellular matrix; TAMs, tumor-associated macrophages; PDOs, patient-derived organoids; AI, artificial intelligence.

## Data Availability

No new data were created or analyzed in this study. Data sharing is not applicable to this article.
